# On the Use of Indian Hemp as an Excitant of Uterine Contractions

**Published:** 1851-10

**Authors:** 


*On the Use of Indian Hemp as an Excitant of Uterine Contractions.*—DR. CHRISTISON, of Edinburgh, considers Indian Hemp, (Cannabis Indica,) to possess a remarkable power of increasing the force of uterine contraction during labor. He reports, in the August number of the *Edinburgh Journal of Medical Science*, some cases in which it was given, with this view, at the Maternity Hospital of Edinburgh. As compared with the action of ergot, that of Indian Hemp presents the following points of difference: "First,—While the effect of ergot does not come on for some considerable time, that of hemp, if it is to appear, is observed within two or three minutes. Secondly,—The action of ergot is of a lasting character, that of hemp is confined to a few pains shortly after its administration. Thirdly,—The action of hemp is more energetic, and perhaps more certainly induced, than that of ergot."

